# Physiological responses and antibiotic-degradation capacity of duckweed (*Lemna aequinoctialis*) exposed to streptomycin

**DOI:** 10.3389/fpls.2022.1065199

**Published:** 2022-12-02

**Authors:** Weijuan Huang, Rui Kong, Lijun Chen, Yuxing An

**Affiliations:** Institute of Nanfan and Seed Industry, Guangdong Academy of Sciences, Guangzhou, China

**Keywords:** antibiotics, duckweed, plant physiology, water contamination, aquatic environment

## Abstract

Aquatic plants are constantly exposed to various water environmental pollutants. Few data on how antibiotics affect duckweed health and its removal ability. The aim of this study was to investigate the impact of streptomycin on the physiological change and uptake capability in duckweed (*Lemna aequinoctialis*) after exposure at different time points (0, 5, 10, 15 and 20 days). Duckweeds were exposed to streptomycin at a range of concentrations (0.1-10 mM). Results indicated that the high streptomycin concentrations (≥1 mM) resulted in a lower duckweed biomass (21.5-41.5%), RGR (0.258-0.336 g d^−1^), decrease in total Chl and increase in carotenoids. Antioxidative enzymes, including CAT (18-42.88 U mg protein^-1^), APX (0.41-0.76 U mg protein^-1^), and SOD (0.52-0.71 U mg protein^-1^) were found to accumulate in the streptomycin groups in comparison to the control group. The significant reduction (72-82%) in streptomycin content at 20 d compared to the control (40-55%) suggested that duckweed has a high ability in removing streptomycin. Transcriptome analysis showed that the secondary metabolic pathways including phenylpropanoid biosynthesis and flavonoid biosynthesis were significantly upregulated in the streptomycin setup compared to the control. Therefore, our findings suggested that duckweed can contribute to the streptomycin degradation, which should be highly recommended to the treatment of aquaculture wastewater and domestic sewage.

## Introduction

In recent years, the wide application of antibiotics has caused great risks to water quality, food and ecological safety after being released into the environment (Kovalakova et al., 2020). Various antibiotics have been found in groundwater, and domestic water that has become a challenge in aquatic ecosystems and could pose serious risks to human health (Jafari Ozumchelouei et al., 2020; Zeng et al., 2022). Although China’s agricultural sector used less than 30,000 tons of antibiotics between 2014 and 2018 (Schoenmakers, 2020), the overall effects on the aquatic ecosystem requires more research. With attentions on environmental protection, there are more and more reports on the effect of antibiotics on the environment and aquatic plant. Sacher et al. (2001) investigated the status and hazards of antibiotic pollution in the environment, analyzed 108 groundwater samples in Germany, and found that 60 kinds of antibiotics in the water samples were detected. Zhang et al. (2017) has also detected quinolones, as well as sulfa and tetracycline in the soil of the Pearl River Delta in China. The impacts of quinotone antibiotics on aquatic environment was investigated, and results showed that quinotone were widely present in the sediments from Yang River and its estuary, and were observed in all five wetland plants (Liu et al., 2020). Tang et al. (2022) studied the evaluation of antibiotics bioaccumulation and trophodynamics in aquatic food webs, and found that metabolic biotransformation plays a significant role in driving biomagnification of antibiotics. Li et al. (2020) reported that a total of 83 target antibiotics were quantified in water and sediment samples collected from the Qinghai Lake, the largest inland lake of China, which suggests the urgent need to investigate the possible long-term enrichment and environmental risks of antibiotics in inland lakes. The findings of Huang et al. (2022) for the first time revealed that the antibiotic synthetic potential in activated sludges could aggravate environmental pollution.

Streptomycin is an aminoglycoside antibiotic with a molecular formula C_21_H_39_N_7_O_12_. Due to its simple availability and cost effectiveness, streptomycin is usually used alone or in combination with other antibiotics to treat bacterial diseases (Lyu et al., 2019). Streptomycin is also widely used in agricultural production to prevent and control plant diseases caused by various pathogenic microorganisms (Walsh et al., 2014). However, its abuse can cause residual drugs to enter the ecosystem and the human body through bio-enrichment and food chain methods, induce various side effects, cause gene mutations, and even cause cancer, which seriously threatens the ecological balance and human health (Yilmaz and Özcengiz, 2017; Wang et al., 2022). Risk assessment of streptomycin residues towards aquatic organisms of various biological processes remains unclear.

Duckweed has 37 species in the world, belonging to five genera including *Lemna, Spirodela, Wolffia, Wolffiella*, and *Landoltia* (Wang et al., 2021). *Lemna aequinoctialis* is a common and widely distributed aquatic plant in China, which has been extensively used in the research of plant biology, aquatic ecotoxicology, and water pollution remediation due to its rapid reproduction, high yield and rich nutritional value (Kummerová et al., 2016; Ekperusi et al., 2019). *Spirodela polyrhiza* has been reported to be able to remove a few antibiotics efficiently (Singh et al., 2018; Singh et al., 2019). After seven days, the medium containing duckweed had significantly lower residual ofloxacin content than the control (Singh et al., 2019). *Lemna paucicostata* removed more than 97% of hydrocarbons from wetlands after 120 days (Ekperusi et al., 2020). Halaimi et al. (2014) found that cadmium and methyl parathion could be removed with both *Lemna gibba* and *Lemna gibba* powder. Baciak et al. (2016) reported that *L. minor* takes up tetracycline in water reservoirs meanwhile the antibiotic significantly affects the duckweed’s metabolism. Most target substances were adequately removed by a continuous-flow *Lemna minor* system with removal rates ranging from 26% (4-methyl-1H-benzotriazole) to 72% (5-chlorobenzotriazole) (Gatidou et al., 2017).

As antibiotic water pollution is gaining prominence as a global issue, the demand for evaluation on its toxicity, mechanism, and remediation has attracted increasing attention. Study on the degradation of streptomycin in water was found consistent with the first-order model in aquatic environments (Shen et al., 2017). Effects of streptomycin on growth of algae indicated that streptomycin is toxic to fresh algae, affects photosynthesis-related gene transcription (Qian et al., 2012). In terms of determining whether streptomycin is harmful to *L. aequinoctialis*, there is currently a lack of information in the scientific literature. Therefore, we carried out a toxicology experiment on duckweed using different concentrations of streptomycin and detected its changes from the physiological and molecular level. The aim of our study was to examine the effects of streptomycin on the duckweed system in terms of antibiotic removal efficiency, growth response, activity of antioxidant enzymes, or relevant physiological and degradation mechanism. Through the evaluation of this study, the potential streptomycin’s toxicological effects on *Lemna aequinoctialis* are expected to be determined.

## Materials and methods

### Sample collection and preparation

The duckweed, *Lemna aequinoctialis* was harvested from a local pond near to the institute and further identified using two DNA barcodes *atpF-atpH* and *psbK-psbI* (Borisjuk et al., 2015). The sterilized duckweeds were cultured on half-strength Schenk & Hildebrandt (SH) basal salt mixture, pH 6, supplemented with 5 g/L glucose. The sterilized duckweed was performed using the method adopted from Huang et al. (2020).

### Evaluation of duckweed toxicity

Different concentrations (0.1, 0.5, 1, 5, and 10 mg L^−1,^ labeled as L5, L4, L3, L2, and L1) of streptomycin in the medium was set as per the methodology of Singh et al. (2019). The 1 g L^-1^ stock solution of streptomycin was made with methanol and 0.1x PBS. The stock solutions were diluted with 0.5x SH medium to creat various streptomycin concentration groups. A half-strength SH medium setup without streptomycin was used as a control. For each concentration, a separate control system (0.5x SH medium + antibiotics without duckweed) was set up to detect changes in photodegradation and hydrolysis other than phytodegradation in antibiotic degradation, which the treatment method was adopted from that described in Singh et al. (2018 and 2019). In a baby jar, we prepared 100 mL of sterile 0.5x SH media (CultureJar™ G9, cat.# C1770; PhytoTechnology Labs, KS, USA) covered with a thin layer of duckweed, and for each concentration of antibiotic with three replicates. The duckweed cultivation was using the method adopted from Huang et al. (2020). The growth period lasted for 30 days, and duckweeds treated with each concentration of antibiotic were harvested respectively at a set time points (0, 5, 10, 15 and 20 days). To maintain a constant growth volume, distilled water was used to replace any evaporation-related water loss during the experiment.

### Duckweed biomass and biochemical analysis

Fresh duckweeds were harvested from the experimental groups every five days, and the change in plant biomass in each group was measured. The RGR (relative growth rate) in all setups at each timeline was measured using the equation RGR = (Dbt_d_ - Dbt_0_)/NA, as described by Verma and Suthar (2014). Where, Dbt_d_ and Dbt0 are the fresh biomass of duckweed recorded every 5 days (5 d, 10d, 15d, and 20d) as well as 0 d, respectively; N is the number of days in the experiment, and A is the baby jar’s medium’s surface area.

The method was used to measure the total chlorophyll and carotenoid content of leaves (Lichtenthaler, 1987). The protein content of harvested duckweed (0.1g) was quantified with a total protein quantitative assay kit. The CAT, SOD, and APX enzyme assay kits were used to quantify catalase, superoxide dismutase, and ascorbate peroxidase, respectively.Analysis of residual streptomycin

Each group’s remaining medium was filtered through a membrane filter of 0.45 m before being collected on days 5, 10, 15, and 20. Streptomycin was detected using HPLC-MS/MS, which was performed using the method adopted from Wang et al. (2019).

### RNA sequencing and analysis

Duckweeds with and without streptomycin treatment were collected on day 5 for RNA sequencing. Total RNA changes after antibiotic treatment were examined using transcriptomic analysis. In order to perform high-throughput sequencing, total RNA was extracted, and the sequences were processed and analyzed followed by Novogene company (Beijing, China). According to the methods of Huang W, et al. (2021), one microgram of total RNA with RIN values above 6.5 was used for next-generation sequencing library preparation. Then, libraries with different indices were multiplexed and loaded on an Illumina HiSeq instrument according to manufacturer’s instructions (Illumina, CA, USA).

### Data analysis

Each treatment’s solution and duckweed samples were used to calculate the mean values and standard deviation (SD) for all three replicates. The exposure levels of antibiotics in solution were calculated using time-weighted average concentrations to express the actual exposure concentrations. All data were compared using SPSS 26.0 (SPSS, Chicago, IL, USA) using repeated measure ANOVA (RMANOVA). The LSD test was used to find significant differences between the analysis variables (*p* < 0.05).

## Results

### Antibiotic-induced changes in biomass and photosynthetic pigments

**Figure 1:**
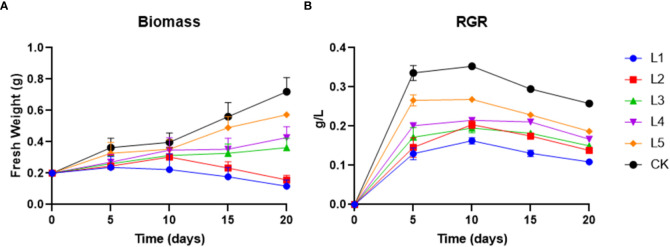
**(A)** Biomass and **(B)** RGR were recorded over four time courses in various streptomycin and control setups. L1, L2, L3, L4, and L5 represented for 10, 5, 1, 0.5, and 0.1 mg L^−1^ concentration of streptomycin, respectively (mean ± SD, n= 3).

As the concentration of streptomycin rises, duckweed’s biomass and photosynthetic pigments tend to decrease in setups compared to the control. The biomass between experimental group and control group at each batch of the experiment was significantly different (*P*<0.05). The harvested duckweed biomass in different treatment setups was found to be ranged between 0.117 g (1 mg/L) and 0.72 g (0.01 mg/L) on day 20 (**Figure 1A**). The experimental group had a greater reduction in biomass exposed to a higher concentration of streptomycin (≥ 5 mg L^−1^) than the control group. At a higher tested concentration (≥ 5 mg L^−1^), L1 and L2 showed a modest but statistically significant inhibition of their RGR from day 10, and they had 41.5% and 21.5% less biomass (day 20) compared to the initial weight (0.2g), respectively. While, the harvested duckweed biomass became increased significantly on day 15 by 63.5%, 76.5%, and 145% in L3, L4 and L5, respectively. The final biomass on day 20 was in the following order: CK > L5 > L4 > L3 > L2 > L1 (**Figure 1A**).

The values of the relative growth rate (RGR) in all streptomycin treatment groups were significantly lower than those of the control (0.258~0.336 g d^−1^) group at the end (**Figure 1B**). For the treatment setups within 10 days, the RGR showed an increment whereas, the RGR was in negative scale in the following ten days. RGR values in each experimental time course showed statistically significant differences between multiple groups. The RGR recorded in L1, L2, L3, L4 and L5 was found to be 0.13, 0.146, 0.172, 0.201 and 0.266 g d^−1^, respectively, compared to the control after 5 days of cultivation (**Figure 1B**).

**Figure 2:**
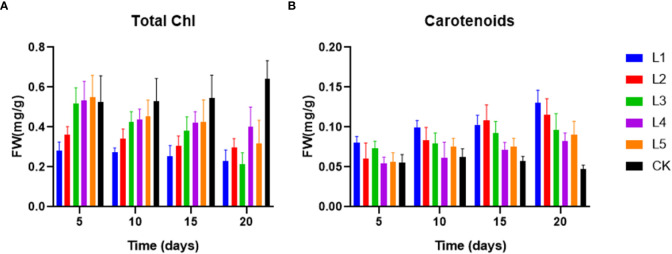
The content of photosynthetic pigments: total Chl **(A)** and carotenoids **(B)** in fronds of *Lemna aequinoctialis* exposed to different concentration of streptomycin in medium.

On day 5, the high dose of streptomycin (10 mg L^-1^) resulted in a significant decrease in total Chl (28.2%), but the subsequent days of cultivation did not show any significant changes (**Figure 2**). Similar to the other four experimental setups, the total Chl content in L1 was trending downward, and the content of total Chl in the end of experiment was in the following order: CK > L4 > L5 > L2 > L1 > L3 (**Figure 2A**). From 0.06 mg g^-1^ in the initial group to 0.13 mg g^-1^ in the group exposed to the highest streptomycin concentration, the carotenoids content significantly increased (**Figure 2B**). The ratio of carotenoids/total Chl increased considerably in setups of streptomycin, suggesting that streptomycin continuously changed the content of photosynthetic pigments.

### Effects of streptomycin on anti-oxidative enzymes in duckweed

**Figure 3:**
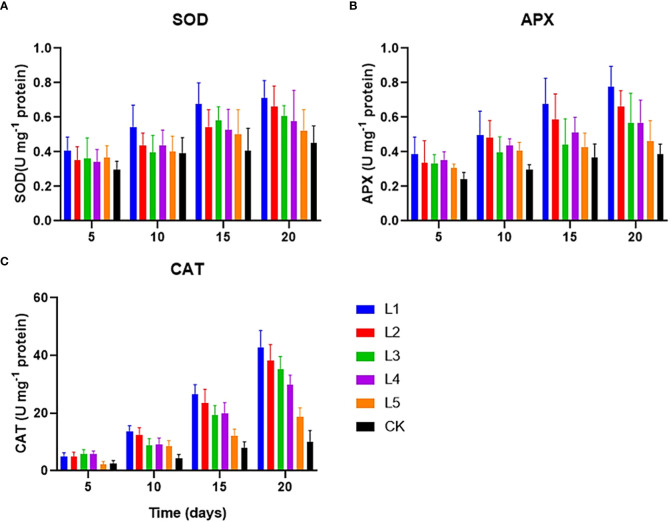
The anti-oxidative enzymes [**(A)** SOD, **(B)**. APX and **(C)** CAT] in *L. aequinoctialis* exposed to a variety of streptomycin concentrations in the medium.

All duckweed setups of various streptomycin concentrations were subjected to a four-time course measurement of the anti-oxidative enzyme activities to determine how streptomycin affected duckweed. The results showed that the streptomycin-treated duckweeds showed an increased activity of SOD, CAT, and APX compared to the control (**Figure 3**). After 10 days of cultivation, the differences of three enzyme activities between different experimental groups became gradually increased. SOD activity increased from 0.52 U mg protein^-1^ to 0.71 U mg protein^-1^ in duckweed setups containing streptomycin by the end of the experiment, indicating a plant anti-oxidative response (**Figure 3A**). Initially, duckweed was significantly inhibited in activity when exposed to high concentrations of streptomycin (≥1 mg L^−1^). As the exposure time increased, the SOD activity in most experimental groups became increased to varying degrees. After exposure for more than two weeks, the SOD activity in duckweed was significantly increased only in a few groups (L1 and L2).

The CAT activity profile showed a different trend. The CAT activity of duckweed tissues did not change with the treatment of 0.1 mg L^-1^ streptomycin. Catalase activity increased gradually during the initial 5 days and afterward grew rapidly from day 5 to day 20 with the exception of the CK and L5 group. CAT activity was the highest (42.88 U mg protein^-1^) in response to 10 mg L^−1^ of the streptomycin on day 20. The CAT showed a significant difference between the various treatments, and multiple comparison tests displayed a significant difference between day 10 to day 20 (**Figure 3B**). Streptomycin can affect the CAT activity of duckweed, and the influence level would be relevant with the concentration of streptomycin and the time of exposure to streptomycin. Especially, when the concentration of streptomycin reached to 1mg/L, the CAT activity of duckweed was significantly inhibited. Diverse streptomycin concentrations significantly affected duckweed’ APX activity, and became increased with higher concentrations of streptomycin (**Figure 3C**). The APX activity continued to increase with the increase of streptomycin dose (L5 < L4 < L3 < L2 < L1, 0.41-0.76 U mg protein^-1^).

### Removal of streptomycin in duckweed reactor

**Figure 4:**
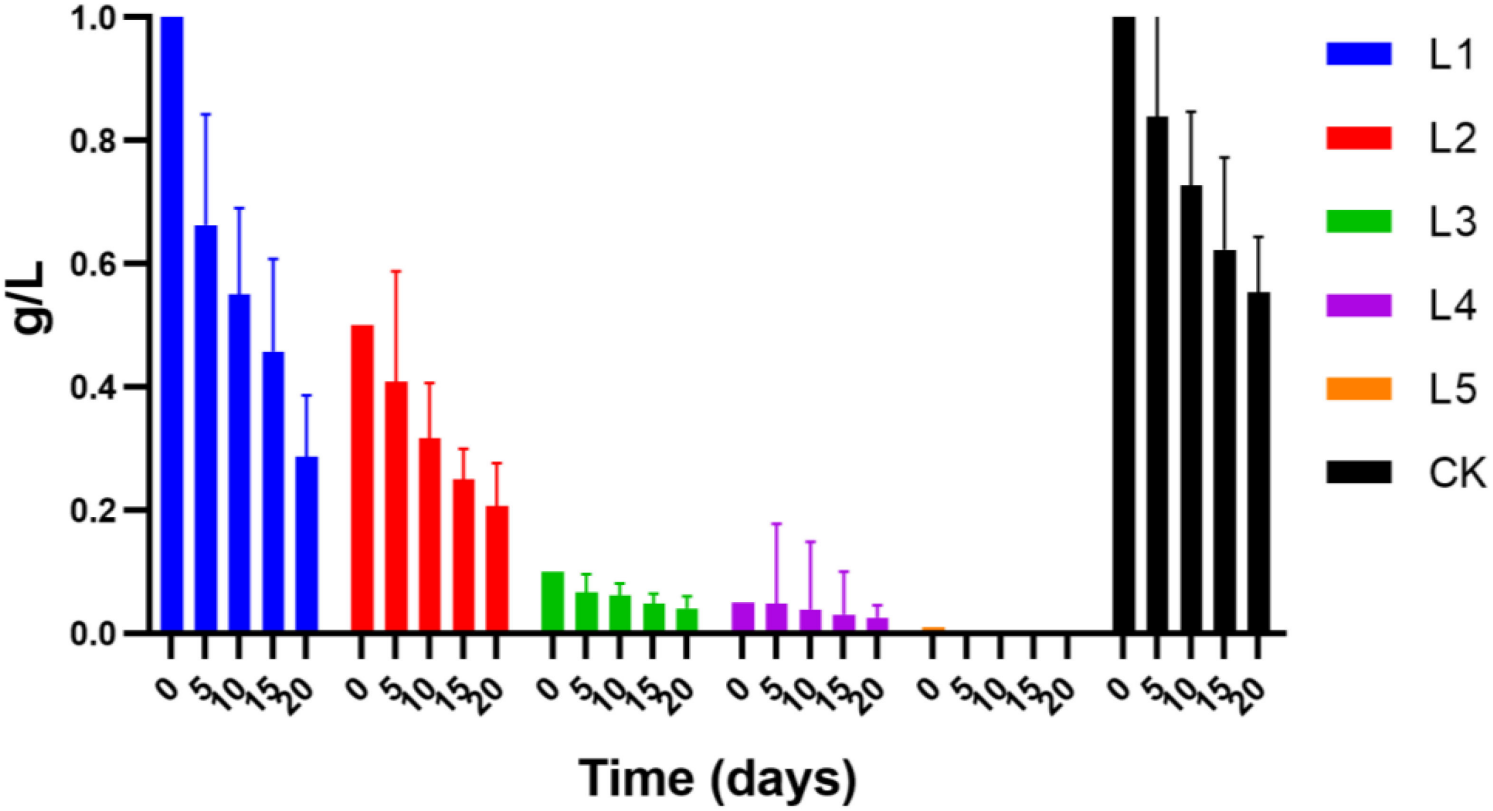
Various concentrations of streptomycin removal by duckweed system.

In order to determine how the antibiotic would behave in the medium when duckweed was present, all batch experiments detected the residual concentration of streptomycin. The results showed that the amount of streptomycin in the duckweed setups decreased by 72% to 82% and in the control by 40% to 55% at the end of the experiment (20 days) (**Figure 4**). In the duckweed setup, the remained concentration of streptomycin at the end of the experiment was L1 > L2 > L3 > L4, indicating that the complete reduction of the antibiotic in the media at a high concentration required more time than that of the low concentration of the antibiotic. The absence of a streptomycin concentration in the L5 group (0.01 mg) indicated that the streptomycin in the solution has been completely degraded. Another possibility could be caused by the detection limit of the instrument used.

### Transcriptomics profiling of duckweed in response to streptomycin

**Figure 5:**
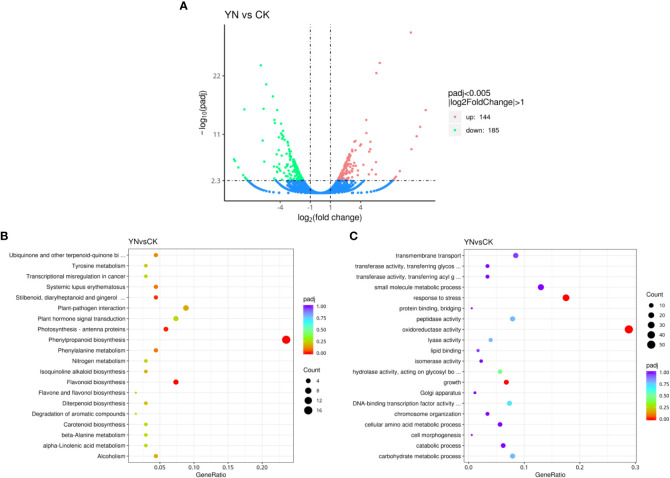
Numbers of differentially expressed genes (DEGs) in duckweed between YN (in response to streptomycin) and CK **(A)**. KEGG **(B)** and GO **(C)** pathway enrichment for DEGs in YN *vs*. CK.

A total of 21,368,136 and 20,568,117 raw reads were obtained from the experimental group (YN) and control group (CK), respectively. Clean reads 20,363,940 and 19,646,041 were retained and used for assembly after low-quality sequences were removed. The RNA-Seq results showed that YN and CK had 329 differentially expressed genes (DEGs). When compared to the CK group, the YN group had a total of 144 genes that were significantly upregulated and 185 genes that were significantly downregulated (**Figure 5A** and **Supplementary Data**). The similar expression patterns observed in nine DEGs (**Supplementary Table 1**) selected at random for qRT-PCR verification confirmed the validity of the transcriptome sequencing data.

Utilizing the GO (Gene Oncology) and KEGG (Kyoto Encyclopedia of Genes and Genomes) databases, we carried out two pathway-based analyses to demonstrate the connection between DEGs and metabolic pathways. An overview of the metabolic changes that occurred in the biological processes of duckweed in response to antibiotics in the aquatic environment was provided by the KEGG enrichment analysis. KEGG enrichment analyses showed that phenylpropanoid biosynthesis, flavonoid biosynthesis, photosynthesis, stillbenoid, and diarylheptanoid were significantly enriched in YN compared to CK (**Figure 5B**). In the YN group, the key genes in the pathway of phenylpropanoid biosynthesis including CYP84A, CYP73A, and CYP98A were significantly downregulated compared to those in the CK group. Similarly, genes in the pathway of flavonoid biosynthesis including CYP73A and CYP98A were significantly downregulated in YN compared to CK. The key genes LHCB1 and LHCB6 in the pathway of photosynthesis were significantly downregulated in YN compared to CK. Functional annotations of DEGs based on the GO pathway analysis showed a number of important metabolic pathways involving oxidoreductase activity, stress response, and growth that were significantly altered in YN compared to CK (**Figure 5C**).

## Discussion

The issues of food safety and environmental pollution brought by the excessive use of antibiotics are becoming increasingly alarming, despite the fact that antibiotics play a crucial role in protecting human health and encouraging the growth of animal husbandry (Ezzariai et al., 2018; Anjali and Shanthakumar, 2019; Zeng et al., 2022). Phytoremediation is an emerging biological technology that aids in the removal of harmful pollutants from water bodies and soil; however, research on phytoremediation requires a comprehensive understanding of processes and mechanisms (Huang D, et al., 2021). Duckweed, as a globally widespread aquatic plant, is attractive for phytoremediation of pollutants in water bodies (Hu et al., 2021; Walsh et al., 2021; Cai et al., 2022). Studies have revealed that duckweed has a great potential in the bioremediation of eutrophic water bodies, heavy metals, antibiotics, insecticides, and organic pollutants (Ceschin et al., 2020; Yang et al., 2020a; Farid et al., 2022; Maldonado et al., 2022). However, whether duckweeds can accelerate the degradation of streptomycin and what are their underlying physiological mechanisms are rarely reported. Based on physiological and molecular biological methods, this study revealed that streptomycin had a severe toxicological effect on the physiological metabolism of duckweed, and found the phytoremediation potential of duckweed on the removal of streptomycin from water body.

The continuous 20-day antibiotic toxicity assessment revealed that streptomycin was toxic to common duckweed at the lowest concentration (0.1 mg L^−1^), resulting in a nearly 15% decrease in growth rate and biomass yield (**Figure 1**). The yield and growth rate of duckweed were both reduced by 45% when streptomycin was applied at a concentration of 10 mg L^-1^. In all of the setups, *Lemna aequinoctialis* did not cause chlorosis to affect the fronds. However, by the end of the experiment, the setups with a higher concentration of streptomycin clearly showed that the fronds had died, suggesting that the antibiotic had a toxic effect on duckweed at a high concentration. This could be explained by the extremely high percentage (93%) of dead mitochondria in duckweed caused by antibiotics in the findings of (Krupka et al., 2021). Maldonado et al. (2022) further revealed that the metabolization of antibiotics occurs in three phases in duckweed which contribute to the removal of antibiotics residues in water. However, the process of antibiotic degradation in duckweed is accompanied by the increase of antioxidant enzymes, slower growth, reduced photosynthesis and other physiological processes (Yang et al., 2020a; Farid et al., 2022), which can be regarded as a cost of adapting to the environmental pollution.

As is reported, streptomycin is an inhibitor of chlorophyll synthesis (Mancinelli et al., 1975). Some abiological factors can stimulate the increase of carotene content, and the function of carotene is to enhance plant resistance under stress conditions. By reducing active reaction centers and preventing electron transport in photosynthetic system II, streptomycin may hinder plant growth (Zhang et al., 2019). In the high dose of streptomycin, the results showed that the total amount of chlorophylls decreased and the amount of carotenoids significantly increased (L1 and L2) (**Figure 2**). In our study, different concentrations of streptomycin can lead to a significant increase in carotenoid content in duckweed, suggesting that duckweed is less resistant to streptomycin stress. Similar results were also found in other aquatic plants, including *Chlorella vulgaris* (Perales-Vela et al., 2016) and *Microcystis aeruginosa* (Qian et al., 2012). Additionally, it has been observed that streptomycin inhibits the synthesis of chlorophyll and carotenoids in the germination of barley seeds (Yaronskaya et al., 2007).

Streptomycin has high water solubility, is easily absorbed by plants, affects the normal physiological metabolism of plants, inhibits plant DNA replication, and thus produces genotoxic effects (Qian et al., 2012). Based on the environmental sensitivity model material duckweed, our study has shown that when given a high dose (0.1 to 10 mg L^-1^) that is appropriate for the environment, streptomycin has a significant negative impact on duckweed’s cellular metabolism and gene expression. According to the transcriptomics data, 329 DEGs were primarily found on the KEGG pathways for phenylpropanoid and flavonoid biosynthesis, which significantly enriched in YN *vs*. CK (**Figure 5**), suggesting that the enriched secondary metabolic pathways may improve duckweed stress resistance. However, there are a lot of relevant genes in plant species that are responsible for the biosynthesis of flavonoids and phenylpropanoid, thus more research is needed to find the target genes and verify their expression. In addition, Hu et al. (2021) clarified the metabolism profiles of the phyllosphere and rhizosphere microbes, providing a fresh perspective on the effects of antibiotics on livestock wastewater through the duckweed system. Therefore, mining the mechanism of plant degradation of pollutants at the molecular level still requires in-depth research.

To the best of our knowledge, there is no published study on how streptomycin affects the duckweed. Regarding the effect of antibiotics on aquatic plants, it is confirmed that antibiotics have a certain threat to aquatic plants, but different aquatic plants have different responses to various types of antibiotics (Harrower et al., 2021; Xu et al., 2021). Research has shown that erythromycin, ciprofloxacin hydrochloride, and sulfamethoxazole all have toxic effects on *Selenastrum capricornutum* (Liu et al., 2011; Kiki et al., 2022). The effects of sulfamethazine, enrofloxacin, and ofloxacin on *Scenedesmus obliquus* were all of low toxicity, but all had certain effects on the physiological indexes of *Scenedesmus obliquus* (Chen et al., 2020; Yang et al., 2020b). Studies have found that the toxicity hazard levels of enrofloxacin and ciprofloxacin hydrochloride to *Isochrysis galbana* are high toxicity and poisoning, respectively (Ge and Deng, 2015). Singh et al. (2018) have found that high concentrations of ofloxacin reduced the biomass, relative root growth rate, protein, and photosynthetic pigment content of duckweed, and increased the activity of antioxidant enzymes in leaves. Meanwhile, duckweed also had a significant degradation effect on ofloxacin. Flumequine and enrofloxacin have inhibitory effects on the plant germination, growth and cell division (Migliore et al., 2000). Therefore, antibiotics not only cause oxidative damage to aquatic plants, but also inhibit the growth and metabolism of aquatic plants.

Not only does duckweed provide food and a habitat for other organisms in the aquatic system, but it also assists in the removal of numerous pollutants from water bodies (Gomes et al., 2020). To understand the risks and effects of antibiotic residues in plant systems, we investigated the effect of streptomycin on duckweed by evaluating the biomass of duckweed, changes of photosynthetic pigments, antioxidant enzymes and other indicators, as well as effects on gene transcription and expression. The findings suggested that the antibiotic had a significant effect on duckweed’s growth, enzyme content, and metabolic pathways when given a high dose of streptomycin; meanwhile, duckweed made greatly contribute to the streptomycin degradation. In conclusion, as one of the producers in the natural ecosystem, aquatic plants play an important role in rebuilding and restoring the aquatic ecological environment. This study, as a case study, can serve as a theoretical foundation for the ecological risk assessment of emerging pollutants in the water environment.

## Data availability statement

The original contributions presented in the study are publicly available. This data can be found here: NCBI, PRJNA896651.

## Author contributions

Conceptualization, WH and YA. Methodology, WH. Investigation, WH, RK and LC. Resources, WH and RK. Data curation, RK. Writing—original draft preparation, WH. Writing—review and editing, WH, LC and YA. Visualization, LC. Project administration, YA. All authors contributed to the article and approved the submitted version.

## Funding

This research was supported by GDAS’Project of Science and Technology Development (2022GDASZH-2022010202), Ministry of Science and Technology of China (G2022030080L), and China Sugar industry Research System (CARS-170306).

## Acknowledgments

We are grateful to Yinglin Lu for assisting sample collection and Yawen Huang for sample test. The work in the Huang Lab was supported by Ministry of Science and Technology of China (G2022030080L).

## Conflict of interest

The authors declare that the research was conducted in the absence of any commercial or financial relationships that could be construed as a potential conflict of interest.

## Publisher’s note

All claims expressed in this article are solely those of the authors and do not necessarily represent those of their affiliated organizations, or those of the publisher, the editors and the reviewers. Any product that may be evaluated in this article, or claim that may be made by its manufacturer, is not guaranteed or endorsed by the publisher.

## References

[B1] Anjali R. Shanthakumar S. (2019). Insights on the current status of occurrence and removal of antibiotics in wastewater by advanced oxidation processes. J. Environ. Manage. 246, 51–62. doi: 10.1016/j.jenvman.2019.05.090 31174030

[B2] Baciak M. Sikorski Ł. Piotrowicz-Cieślak A. I. Adomas B. (2016). Content of biogenic amines in *Lemna minor* (common duckweed) growing in medium contaminated with tetracycline. Aquat Toxicol. 180, 95–102. doi: 10.1016/j.aquatox.2016.09.007 27684602

[B3] Borisjuk N. Chu P. Gutierrez R. Zhang H. Acosta K. Friesen N.. (2015). Assessment, validation and deployment strategy of a two-barcode protocol for facile genotyping of duckweed species. Plant Biol. (Stuttgart Germany) 17 Suppl 1, 42–49. doi: 10.1111/plb.12229 25115915

[B4] Cai X. Y. Xu M. Zhu Y. X. Shi Y. Wang H. W. (2022). Removal of dinotefuran, thiacloprid, and imidaclothiz neonicotinoids in water using a novel *Pseudomonas monteilii* FC02-duckweed (*Lemna aequinoctialis*) partnership. Front. Microbiol. 13. doi: 10.3389/fmicb.2022.906026 PMC9218866 35756054

[B5] Ceschin S. Crescenzi M. Iannelli M. A. (2020). Phytoremediation potential of the duckweeds *Lemna minuta* and *Lemna minor* to remove nutrients from treated waters. Environ. Sci. pollut. Res. Int. 27, 15806–15814. doi: 10.1007/s11356-020-08045-3 32088823

[B6] Chen Q. Zhang L. Han Y. Fang J. Wang H. (2020). Degradation and metabolic pathways of sulfamethazine and enrofloxacin in *Chlorella vulgaris* and *Scenedesmus obliquus* treatment systems. Environ. Sci. pollut. Res. Int. 27, 28198–28208. doi: 10.1007/s11356-020-09008-4 32415445

[B7] Ekperusi A. O. Nwachukwu E. O. Sikoki F. D. (2020). Assessing and modelling the efficacy of *Lemna paucicostata* for the phytoremediation of petroleum hydrocarbons in crude oil-contaminated wetlands. Sci. Rep. 22, 8489. doi: 10.1038/s41598-020-65389-z PMC7244528 32444776

[B8] Ekperusi A. O. Sikoki F. D. Nwachukwu E. O. (2019). Application of common duckweed (*Lemna minor*) in phytoremediation of chemicals in the environment: State and future perspective. Chemosphere. 223, 285–309. doi: 10.1016/j.chemosphere.2019.02.025 30784736

[B9] Ezzariai A. Hafidi M. Khadra A. Aemig Q. El Fels L. Barret M.. (2018). Human and veterinary antibiotics during composting of sludge or manure: Global perspectives on persistence, degradation, and resistance genes. J. Hazard Mater. 359, 465–481. doi: 10.1016/j.jhazmat.2018.07.092 30071464

[B10] Farid M. Sajjad A. Asam Z. Zubair M. Rizwan M. Abbas M.. (2022). Phytoremediation of contaminated industrial wastewater by duckweed (*Lemna minor* l.): Growth and physiological response under acetic acid application. Chemosphere 304, 135262. doi: 10.1016/j.chemosphere.2022.135262 35688199

[B11] Gatidou G. Oursouzidou M. Stefanatou A. Stasinakis A. S. (2017). Removal mechanisms of benzotriazoles in duckweed *Lemna minor* wastewater treatment systems. Sci. Total Environ. 596-597, 12–17. doi: 10.1016/j.scitotenv.2017.04.051 28412566

[B12] Ge L. Deng H. (2015). Degradation of two fluoroquinolone antibiotics photoinduced by Fe(III)-microalgae suspension in an aqueous solution. Photochem. Photobiol. Sci. 14, 693–699. doi: 10.1039/c3pp50149c 25590051

[B13] Gomes M. P. Moreira Brito J. C. Cristina Rocha D. Navarro-Silva M. A. Juneau P. (2020). Individual and combined effects of amoxicillin, enrofloxacin, and oxytetracycline on *Lemna minor* physiology. Ecotoxicol Environ. Saf. 203, 111025. doi: 10.1016/j.ecoenv.2020.111025 32888593

[B14] Halaimi F. Z. Kellali Y. Couderchet M. Semsari S. (2014). Comparison of biosorption and phytoremediation of cadmium and methyl parathion, a case-study with live *Lemna gibba* and *Lemna gibba* powder. Ecotoxicol Environ. Saf. 105, 112–120. doi: 10.1016/j.ecoenv.2014.02.002 24815048

[B15] Harrower J. McNaughtan M. Hunter C. Hough R. Zhang Z. Helwig K. (2021). Chemical fate and partitioning behavior of antibiotics in the aquatic environment-a review. Environ. Toxicol. Chem. 40, 3275–3298. doi: 10.1002/etc.5191 34379810

[B16] Huang W. Gilbert S. Poulev A. Acosta K. Lebeis S. Long C. L.. (2020). Host-specific and tissue-dependent orchestration of microbiome community structure in traditional rice paddy ecosystems. Plant Soil 452, 379–395. doi: 10.1007/s11104-020-04568-3

[B17] Huang W. Sun D. Wang R. An Y. (2021). Integration of transcriptomics and metabolomics reveals the responses of sugar beet to continuous cropping obstacle. Front. Plant Sci. 12. doi: 10.3389/fpls.2021.711333 PMC8578061 34777408

[B18] Huang D. Xiao R. Du L. Zhang G. Yin L. Deng R.. (2021). Phytoremediation of poly- and perfluoroalkyl substances: A review on aquatic plants, influencing factors, and phytotoxicity. J. Hazard Mater. 418, 126314. doi: 10.1016/j.jhazmat.2021.126314 34329029

[B19] Huang Y. Zou K. Qing T. Feng B. Zhang P. (2022). Metagenomics and metatranscriptomics analyses of antibiotic synthesis in activated sludge. Environ. Res. 213, 113741. doi: 10.1016/j.envres.2022.113741 35750126

[B20] Hu H. Li X. Wu S. Lou W. Yang C. (2021). Effects of long-term exposure to oxytetracycline on phytoremediation of swine wastewater *via* duckweed systems. J. Hazard Mater. 414, 125508. doi: 10.1016/j.jhazmat.2021.125508 34030403

[B21] Jafari Ozumchelouei E. Hamidian A. H. Zhang Y. Yang M. (2020). Physicochemical properties of antibiotics: A review with an emphasis on detection in the aquatic environment. Water Environ. Res. 92, 177–188. doi: 10.1002/wer 31505071

[B22] Kiki C. Ye X. Li X. Adyari B. Hu A. Qin D.. (2022). Continuous antibiotic attenuation in algal membrane photobioreactor: Performance and kinetics. J. Hazard Mater. 434, 128910. doi: 10.1016/j.jhazmat.2022.128910 35452987

[B23] Kovalakova P. Cizmas L. McDonald T. J. Marsalek B. Feng M. Sharma V. K. (2020). Occurrence and toxicity of antibiotics in the aquatic environment: A review. Chemosphere 251, 126351. doi: 10.1016/j.chemosphere.2020.126351 32443222

[B24] Krupka M. Michalczyk D. J. Žaltauskaitė J. Sujetovienė G. Głowacka K. Grajek H.. (2021). Physiological and biochemical parameters of common duckweed *Lemna minor* after the exposure to tetracycline and the recovery from this stress. Molecules. 26, 6765. doi: 10.3390/molecules26226765 34833856 PMC8625026

[B25] Kummerová M. Zezulka Š. Babula P. Tříska J. (2016). Possible ecological risk of two pharmaceuticals diclofenac and paracetamol demonstrated on a model plant *Lemna minor* . J. Hazard Mater. 302, 351–361. doi: 10.1016/j.jhazmat.2015.09.057 26476323

[B26] Li S. Kuang Y. Hu J. You M. Guo X. Gao Q.. (2020). Enrichment of antibiotics in an inland lake water. Environ. Res. 190, 110029. doi: 10.1016/j.envres.2020.110029 32795452

[B27] Lichtenthaler H. K. (1987). Chlorophylls and carotenoids: Pigments of photosynthetic biomembranes. Method Enzymol. 148, 350–382. doi: 10.1016/0076-6879(87)48036-1

[B28] Liu B. Y. Nie X. P. Liu W. Q. Snoeijs P. Guan C. Tsui M. T. (2011). Toxic effects of erythromycin, ciprofloxacin and sulfamethoxazole on photosynthetic apparatus in *Selenastrum capricornutum* . Ecotoxicol Environ. Saf. 74, 1027–1035. doi: 10.1016/j.ecoenv.2011.01.022 21353704

[B29] Liu K. Zhang D. Xiao X. Cui L. Zhang H. (2020). Occurrence of quinotone antibiotics and their impacts on aquatic environment in typical river-estuary system of jiaozhou bay, China. Ecotoxicol Environ. Saf. 190, 109993. doi: 10.1016/j.ecoenv.2019.109993 31869715

[B30] Lyu Q. Bai K. Kan Y. Jiang N. Thapa S. P. Coaker G.. (2019). Variation in streptomycin resistance mechanisms in *Clavibacter michiganensis* . Phytopathology. 109, 1849–1858. doi: 10.1094/PHYTO-05-19-0152-R 31334679

[B31] Maldonado I. Moreno Terrazas E. G. Vilca F. Z. (2022). Application of duckweed (*Lemna* sp.) and water fern (*Azolla* sp.) in the removal of pharmaceutical residues in water: State of art focus on antibiotics. Sci. Total Environ. 838, 156565. doi: 10.1016/j.scitotenv.2022.156565 35690203

[B32] Mancinelli A. L. Yang C. P. Lindquist P. Anderson O. R. Rabino I. (1975). Photocontrol of anthocyanin synthesis: III. the action of streptomycin on the synthesis of chlorophyll and anthocyanin. Plant Physiol. 55, 251–257. doi: 10.1104/pp.55.2.251 16659061 PMC541594

[B33] Migliore L. Cozzolino S. Fiori M. (2000). Phytotoxicity to and uptake of flumequine used in intensive aquaculture on the aquatic weed, *Lythrum salicaria* l. Chemosphere. 40, 741–750. doi: 10.1016/s0045-6535(99)00448-8 10705552

[B34] Perales-Vela H. V. García R. V. Gómez-Juárez E. A. Salcedo-Álvarez M. O. Cañizares-Villanueva R. O. (2016). Streptomycin affects the growth and photochemical activity of the alga *Chlorella vulgaris* . Ecotoxicol Environ. Saf. 132, 311–317. doi: 10.1016/j.ecoenv.2016.06.019 27344399

[B35] Qian H. Li J. Pan X. Sun Z. Ye C. Jin G.. (2012). Effects of streptomycin on growth of algae *Chlorella vulgaris* and microcystis aeruginosa. Environ. Toxicol. 27, 229–237. doi: 10.1002/tox.20636 20725941

[B36] Sacher F. Lange F. T. Brauch H. J. Blankenhorn I. (2001). Pharmaceuticals in groundwaters analytical methods and results of a monitoring program in Baden-württemberg, Germany. J. Chromatogr A. 938, 199–210. doi: 10.1016/s0021-9673(01)01266-3 11771839

[B37] Schoenmakers K. (2020). How China is getting its farmers to kick their antibiotics habit. Nature 586, S60–S62. doi: 10.1038/d41586-020-02889-y

[B38] Shen Y. Zhao W. Zhang C. Shan Y. Shi J. (2017). Degradation of streptomycin in aquatic environment: kinetics, pathway, and antibacterial activity analysis. Environ. Sci. pollut. Res. Int. 24, 14337–14345. doi: 10.1007/s11356-017-8978-5 28429270

[B39] Singh V. Pandey B. Suthar S. (2018). Phytotoxicity of amoxicillin to the duckweed *Spirodela polyrhiza*: Growth, oxidative stress, biochemical traits and antibiotic degradation. Chemosphere. 201, 492–502. doi: 10.1016/j.chemosphere.2018.03.010 29529576

[B40] Singh V. Pandey B. Suthar S. (2019). Phytotoxicity and degradation of antibiotic ofloxacin in duckweed (*Spirodela polyrhiza*) system. Ecotoxicol Environ. Saf. 179, 88–95. doi: 10.1016/j.ecoenv.2019.04.018 31026754

[B41] Tang J. Zhang J. Su L. Jia Y. Yang Y. (2022). Bioavailability and trophic magnification of antibiotics in aquatic food webs of pearl river, China: Influence of physicochemical characteristics and biotransformation. Sci. Total Environ. 820, 153285. doi: 10.1016/j.scitotenv.2022.153285 35066051

[B42] Verma R. Suthar S. (2014). Synchronized urban wastewater treatment and biomass production using duckweed *Lemna gibba* l. Ecol. Eng. 64, 337–343. doi: 10.1016/j.ecoleng.2013.12.055

[B43] Walsh É. Kuehnhold H. O'Brien S. Coughlan N. E. Jansen M. (2021). Light intensity alters the phytoremediation potential of *Lemna minor* . Environ. Sci. pollut. Res. Int. 28, 16394–16407. doi: 10.1007/s11356-020-11792-y 33387327

[B44] Walsh F. Smith D. P. Owens S. M. Duffy B. Frey J. E. (2014). Restricted streptomycin use in apple orchards did not adversely alter the soil bacteria communities. Front. Microbiol. 4. doi: 10.3389/fmicb.2013.00383 PMC3908321 24550889

[B45] Wang K. T. Hong M. C. Wu Y. S. Wu T. M. (2021). Agrobacterium-mediated genetic transformation of Taiwanese isolates of *Lemna aequinoctialis* . Plants (Basel). 10, 1576. doi: 10.3390/plants10081576 34451621 PMC8401387

[B46] Wang S. Wang H. Du T. Bu T. Xu J. Liu S.. (2022). Multiplex immunochromatographic platform based on crystal violet tag for simultaneous detection of streptomycin and chloramphenicol. Food Chem. 393, 133351. doi: 10.1016/j.foodchem.2022.133351 35689929

[B47] Wang Z. Xie C. Yeung S. Wang J. Chow M. (2019). Development of a simple and rapid HPLC-MS/MS method for quantification of streptomycin in mice and its application to plasma pharmacokinetic studies. BioMed. Chromatogr. 33, e4408. doi: 10.1002/bmc.4408 30324683

[B48] Xu L. Zhang H. Xiong P. Zhu Q. Liao C. Jiang G. (2021). Occurrence, fate, and risk assessment of typical tetracycline antibiotics in the aquatic environment: A review. Sci. Total Environ. 753, 141975. doi: 10.1016/j.scitotenv.2020.141975 33207448

[B49] Yang L. Chen Y. Shi L. Yu J. Yao J. Sun J.. (2020a). Enhanced cd accumulation by graphene oxide (GO) under cd stress in duckweed. Aquat Toxicol. 229, 105579. doi: 10.1016/j.aquatox.2020.105579 33075615

[B50] Yang L. Ren L. Tan X. Chu H. Chen J. Zhang Y.. (2020b). Removal of ofloxacin with biofuel production by oleaginous microalgae *Scenedesmus obliquus* . Bioresour Technol. 315, 123738. doi: 10.1016/j.biortech.2020.123738 32659423

[B51] Yaronskaya E. B. Gritskevich E. R. Trukhanovets N. L. Averina N. G. (2007). Effect of kinetin on early stages of chlorophyll biosynthesis in streptomycin-treated barley seedlings. Russ. J. Plant Physiol. 54, 388–395. doi: 10.1134/S1021443707030144

[B52] Yilmaz Ç. Özcengiz G. (2017). Antibiotics: Pharmacokinetics, toxicity, resistance and multidrug efflux pumps. Biochem. Pharmacol. 133, 43–62. doi: 10.1016/j.bcp.2016.10.005 27765485

[B53] Zeng Y. Chang F. Liu Q. Duan L. Li D. Zhang H. (2022). Recent advances and perspectives on the sources and detection of antibiotics in aquatic environments. J. Anal. Methods Chem., 2022, 5091181. doi: 10.1155/2022/5091181 35663459 PMC9159860

[B54] Zhang H. B. Dong C. C. Yang Y. J. Fu J. K. Liu L. He X. Y.. (2019). The toxic effect of streptomycin on the growth and photosynthesis of nostoc using the chlorophyll fluorescence analysis. Acta Hydrobiologica Sin. 43, 664–669.

[B55] Zhang R. Zhang R. Zou S. Yang Y. Li J. Wang Y.. (2017). Occurrence, distribution and ecological risks of fluoroquinolone antibiotics in the dongjiang river and the beijiang river, pearl river delta, south China. Bull. Environ. Contam Toxicol. 99, 46–53. doi: 10.1007/s00128-017-2107-5 28555337

