# Supplementary material for: Physiological responses and antibiotic-degradation capacity of duckweed (Lemna aequinoctialis) exposed to streptomycin
Source: Front Plant Sci. 2022 Dec 2;13:1065199. doi: 10.3389/fpls.2022.1065199 (PMC9774486; doi:10.3389/fpls.2022.1065199)
Supplement: Supplementary file 2 [file Table_2.docx]

**Supplementary Table 1 Specific primers of RT-qPCR amplification**

| Gene ID | Gene name | Primer(5'-3') |
| --- | --- | --- |
|  |  |  |
| gene-LOC104904096 | NADPH dehydrogenase | F-TCCTGGCTCTGCTTCAACAACC  R-GACGACGGTGACCTTCTTCTCC |
| gene-LOC104904819 | Cytochrome C | F-ATGGCACACTGAAATCGCATCG  R-TGGCTCCTCCTTCTTTGGTTCC |
| gene-LOC104887841 | NAD(P)H-nitrite reductase | F-GCCGCTGGATTTGATGCTGATT  R-TGCACCAACACCGACAACAAC |
| gene-LOC109133531 | Glycosyl transferases | F-GCCTCACCGACTCAGACACCTT  R-GATTTGGCGGCGGAGGAAGT |
| gene-LOC104883503 | Glutamine synthetase | F-GCTATTGGAGCGGACAAATCGT  R-CCACTGACCAGGCATCACTTCT |
| gene-LOC104888263 | Glutamate synthase | F-AGAGCAAGTGGGTGGCAAAGG  R-GGGCAACCGCATTTCTTCCAAT |
| gene-LOC104888710 | Glutamate dehydrogenase | F-ACAACGAGATTCGCCGATTCTG  R-CGTCCGCCAACACCAATATCAC |
| gene-LOC104887645 | Superoxide dismutase | F-TCACCACCAGAAGCACCATCAA  R-GGACAACCTTGGAAGCATCCCT |
|  | β-actin | F-GGGGATGAAGCACAGTCCAA  R-GCCGTGGTTGTGAAGGAGTA |
|  | GAPDH | F-GTGGTTTCCAACGCATCCTG  R-GCTCTCCAGTCCTTACCTCCCT |
